# The transcription factor RhMYB17 regulates the homeotic transformation of floral organs in rose (*Rosa hybrida*) under cold stress

**DOI:** 10.1093/jxb/erae099

**Published:** 2024-03-07

**Authors:** Tuo Yang, Yi Wang, Yuqi Li, Shangyi Liang, Yunyao Yang, Ziwei Huang, Yonghong Li, Junping Gao, Nan Ma, Xiaofeng Zhou

**Affiliations:** Beijing Key Laboratory of Development and Quality Control of Ornamental Crops, Department of Ornamental Horticulture, China Agricultural University, Beijing, China; Beijing Key Laboratory of Development and Quality Control of Ornamental Crops, Department of Ornamental Horticulture, China Agricultural University, Beijing, China; Beijing Key Laboratory of Development and Quality Control of Ornamental Crops, Department of Ornamental Horticulture, China Agricultural University, Beijing, China; Beijing Key Laboratory of Development and Quality Control of Ornamental Crops, Department of Ornamental Horticulture, China Agricultural University, Beijing, China; Beijing Key Laboratory of Development and Quality Control of Ornamental Crops, Department of Ornamental Horticulture, China Agricultural University, Beijing, China; Beijing Key Laboratory of Development and Quality Control of Ornamental Crops, Department of Ornamental Horticulture, China Agricultural University, Beijing, China; School of Food and Drug, Shenzhen Polytechnic University, Shenzhen, China; Beijing Key Laboratory of Development and Quality Control of Ornamental Crops, Department of Ornamental Horticulture, China Agricultural University, Beijing, China; Beijing Key Laboratory of Development and Quality Control of Ornamental Crops, Department of Ornamental Horticulture, China Agricultural University, Beijing, China; Beijing Key Laboratory of Development and Quality Control of Ornamental Crops, Department of Ornamental Horticulture, China Agricultural University, Beijing, China; University College Dublin, Ireland

**Keywords:** ABCDE model, APETALA 2, homeotic transformation of floral organs, low temperature, MYB, *Rosa hybrida*

## Abstract

Low temperatures affect flower development in rose (*Rosa hybrida*), increasing petaloid stamen number and reducing normal stamen number. We identified the low-temperature-responsive R2R3-MYB transcription factor RhMYB17, which is homologous to Arabidopsis MYB17 by similarity of protein sequences. *RhMYB17* was up-regulated at low temperatures, and *RhMYB17* transcripts accumulated in floral buds. Transient silencing of *RhMYB17* by virus-induced gene silencing decreased petaloid stamen number and increased normal stamen number. According to the ABCDE model of floral organ identity, class A genes *APETALA 1* (*AP1*) and *AP2* contribute to sepal and petal formation. Transcription factor binding analysis identified RhMYB17 binding sites in the promoters of rose *APETALA 2* (*RhAP2*) and *APETALA 2-LIKE* (*RhAP2L*). Yeast one-hybrid assays, dual-luciferase reporter assays, and electrophoretic mobility shift assays confirmed that RhMYB17 directly binds to the promoters of *RhAP2* and *RhAP2L*, thereby activating their expression. RNA sequencing further demonstrated that RhMYB17 plays a pivotal role in regulating the expression of class A genes, and indirectly influences the expression of the class C gene. This study reveals a novel mechanism for the homeotic transformation of floral organs in response to low temperatures.

## Teaser

Under cold stress, the transformation from stamens to petals in the rose flower is mediated by the transcription factor RhMYB17, which activates expression of *APETALA 2* and *APETALA 2-LIKE* .

## Introduction

The ABCDE model for floral development describes the functions of A, B, C, D, and E genes (Coen and Meyerowitz, 1991; Thei and Saedler, 2001; Krizek and Fletcher, 2005). According to this model, the flower comprises four regions, known as ‘whorls’ of floral organs, wherein combinations of class A, B, C, D, and E homeotic genes work in concert to determine floral organ identity (Theißen *et al*., 2016). In whorl 1, class A genes (*APETALA 1* (*AP1*) and *APETALA 2* (*AP2*)) solely determine sepal identity; in whorl 2, class A and B genes (*AP3* and *PISTILLATA* (*PI*)) collaboratively specify petal identity; in whorl 3, class B and C genes (*AGAMOUS* (*AG*)) define stamen identity; and in whorl 4, a class C gene exclusively determines carpel identity. Class D genes play a crucial role in establishing ovule identity, whereas ectopic expression of these genes leads to the formation of ovules in atypical locations such as sepals and petals (Kater *et al*., 2006). Class E genes affect the development of all floral organs (Robles and Pelaz, 2004; Yan *et al*., 2016). In Arabidopsis, all class A, B, C, D, and E genes except the class A gene *AP2* are MIKC^C^-type MADS box genes.

Among genes involved in floral organ development, *AP2*, *AP2L*, and *AG* play a key role in floral determinacy and also serve as a major regulator of floral organ trait specification. The *RhAP2* gene is thought to function as the major regulator of petal number in roses (*Rosa hybrida*) (Hibrand Saint-Oyant *et al*., 2018). *APETALA 2-like* gene (*AP2L*), a member of the Target Of EAT-type (TOE-type) subfamily, is involved in regulating the double-flower phenotype of roses (François *et al*., 2018). *AG* determines stamen identity in whorl 3 and carpel identity in whorl 4 (Bowman *et al*., 1991). In the Arabidopsis *ag* mutant, stamens and carpels are replaced by petals and sepals, respectively. *AP2* restricts *AG* expression to the inner two whorls of the flower (Drews *et al*., 1991; Jofuku *et al*., 1994). In *ap2* loss-of-function mutants, ectopic expression of *AG* in the outer two whorls leads to the transformation of sepals and petals into stamens (Bowman *et al*., 1991; Drews *et al*., 1991). AtAP2 promotes the maintenance of floral stem cell fate, not by inhibiting *AtAG* transcription, but by antagonizing *AtAG* activity in the floral center (Huang *et al*., 2017). *AP2* and *AG* exhibit antagonistic expression patterns, restricting each other’s activities to specific regions within the floral meristem to govern the homeotic transformation of floral organs. In peach (*Prunus persica*), the restricted expression of the class C gene is primarily due to the activities of miRNA172, which is also associated with genes of the euAP2 family (Gattolin *et al*., 2020). Exogenously expressing *RcAP2* from *Rosa chinensis* in Arabidopsis induces the transformation of stamens into petals, thereby increasing petal number. Conversely, silencing *RcAP2* reduces petal number (Han *et al*., 2018). A transposable element has been inserted into an intron of *RcAP2L*, resulting in the creation of a miRNA172-resistant *RcAP2L* variant. This leads to the stable expression of *RhAP2L*, facilitating the double flower phenotype in roses (Chen, 2004; François *et al*., 2018). Similar observations have been made in kiwifruit (*Actinidia deliciosa*) (Varkonyi-Gasic *et al*., 2012).

The initiation and determination of floral organs are also controlled by low temperature. Early studies found that 5 °C conditions promote the formation of secondary flowers in the center of carnation (*Dianthus caryophyllus*) flowers, resulting in an increase in the total number of petals (Garrod and Harris, 1974). Silencing of the class C gene *RhAG* increases petal number in rose (Ma *et al*., 2015). Under low-temperature conditions, the overall spatial accumulation of *RhAG* transcripts in floral buds is significantly reduced and the promoter of *RhAG* is hypermethylated (Ma *et al*., 2015). However, the molecular mechanisms by which low temperatures affect floral organ development have not been characterized.

Numerous transcription factors, including MYB, WRKY, bZIP, and NAC, function in plant responses to environmental stress. The MYB transcription factor family is divided into four categories based on the number of MYB domains in each protein: 1R-, R2R3-, 3R-, and 4R-MYB (Rogers and Campbell, 2004). R2R3-MYB proteins are unique to plants and represent the most abundant MYB proteins. Various R2R3-MYB transcription factors actively participate in plant growth and development, secondary metabolism, and biotic and abiotic stress responses (Lotkowska *et al*., 2015; Li *et al*., 2019; Liu *et al*., 2021). R2R3-MYB transcription factors have roles in cold resistance in various plant species, including Arabidopsis, maize (*Zea mays*), rice (*Oryza sativa*), apple (*Malus domestica*), and rose. In Arabidopsis, various R2R3-MYB transcription factors function in stress responses, such as AtMYB15, AtMYB30, AtMYB44, AtMYB96, and AtMYB108 (Dubos *et al*., 2010). AtMYB15 interacts with INDUCER OF C-REPEAT BINDING FACTOR EXPRESSION 1 (ICE1) and binds to the MYB recognition sequences in the promoters of C-repeat binding factor (*CBF*) genes to enhance cold tolerance (Agarwal *et al*., 2006). In maize, the R2R3-MYB transcription factor ZmMYB31 positively regulates *CBF* gene expression and enhances resistance to low temperature (Li *et al*., 2019). In rice, OsMYB30 responds to low-temperature stress by negatively regulating the expression of downstream low-temperature-responsive genes (Lv *et al*., 2017). The expression of the R2R3-MYB transcription factor gene *MdMYB73* is induced by cold stress in apple. Overexpressing *MdMYB73* significantly improves cold resistance in transgenic apple callus and Arabidopsis (Zhang *et al*., 2017). Transcriptomic analysis of *Rosa multiflora* leaves under different temperature treatments (25, 4, and −20 °C) revealed that genes encoding AP2/ethylene response factors and MYB transcription factors actively participated in the response to cold treatment (Zhang *et al*., 2016). In purple kale (*Brassica oleracea*), low temperature strongly promotes anthocyanin biosynthesis via the up-regulation of *BoPAP1*, thereby enhancing cold tolerance (Zhang *et al*., 2012). The R2R3-MYB transcription factors MdMYB124 and MdMYB88 positively regulate the expression of cold-responsive genes in apple to improve tolerance to cold stress (Xie *et al*., 2017). In summary, many studies have revealed that R2R3-MYB transcription factors play essential roles in plant responses to low temperature.

In addition, many MYB transcription factors play a role in regulating the development of the shoot apical meristem and floral organs, such as AtMYB13, AtMYB16, AtMYB17, AtMYB56, and AtMYB117 in Arabidopsis. AtMYB13 operates as a key component within a regulatory network that governs the establishment of the shoot system by modulating the function of the apical meristem (Kirik *et al*., 1998). AtMYB16 is involved in the regulation of epidermal cell morphogenesis in petals (Baumann *et al*., 2007). AtMYB56 acts as a negative regulator of flowering by repressing *FT* transcription (Chen *et al*., 2015). AtMYB117/LOF1 facilitates the development of floral organs and initiates ovule outgrowth (Gomez *et al.,* 2011). AtMYB17/LMI2 can influence flower development by repressing *AtANT* (Zhang *et al*., 2009). AtMYB17 orchestrates the meristem identity transition from vegetative growth to flowering, functioning subsequent to *AtLFY* and preceding *AtAP1*. It directly activates *AtAP1* to endorse floral fate. In conjunction with AtLFY and AtAP1, it likely forms a regulatory framework facilitating a transition in meristem identity (Pastore *et al*., 2011). Previous studies have shown that RhMYB123, a member of the MYB transcription factor family, regulates stamen–petal organ specification in rose (Li *et al*., 2022). However, little is known about the effects of these MYB transcription factors on the homeotic transformation of floral organs at low temperature.

In this study, we discovered changes indicative of the homeotic transformation of floral organs from stamens to petals in rose under low-temperature conditions. The R2R3-MYB transcription factor gene *RhMYB17* exhibited increased expression at low temperatures. We demonstrate that RhMYB17 functions as a transcriptional activator of *RhAP2* and *RhAP2L*, thereby specifying stamen–petal homeotic transformation at low temperature.

## Materials and methods

### Plant materials and treatments

Rose (*Rosa hybrida* cv. ‘Samantha’) and *Nicotiana benthamiana* plants were grown as described previously (Zhang *et al*., 2019). Rose stems were cultured in Murashige and Skoog (MS) medium supplemented with 1.0 mg l^−1^ 6-benzyl aminopurine and 0.05 mg l^−1^ 1-naphthaleneacetic acid (NAA) for 30 d in a culture room at 22 ± 1 °C under a 16 h light–8 h dark photoperiod with light intensity of 100–120 μmol m^−2^ s^−1^ (Wu *et al*., 2017). Rose cuttings were cultured in half-strength MS medium supplemented with 0.1 mg l^−1^ NAA for 20–30 d to induce root formation. Rooted cuttings were transferred to pots containing peat moss: vermiculite (1:1) and grown in a culture room at 22 ± 1 °C at a relative humidity of ~60% under a 16 h light–8 h dark photoperiod with light intensity of 100–120 μmol m^−2^ s^−1^. When the floral buds appeared and reached a length of more than 2 mm (stage 4), the plants were incubated at 4 °C (low temperature). After the low-temperature treatment, the roses were returned to normal conditions. Once the roses were fully opened, the number of floral organs were counted. Normal petals are defined by their petal cells growing symmetrically, and they are often the outer whorl petals. In contrast, petaloid stamens are characterized by asymmetrical petal tissue and the retention of some stamen characteristics.

Tobacco was planted in pots and grown in a growth chamber under the same growth conditions.

### *In situ* hybridization

Processing of floral bud samples and *in situ* hybridization were performed as previously described (Zhang *et al*., 2007; Ma *et al*., 2008, 2015; Chen *et al*., 2021). *In situ* hybridization probes were synthesized by PCR amplification of *RhMYB17*, *RhAP2*, *RhAP2L*, and *RhAG* cDNAs using gene-specific primers containing T7 and SP6 RNA polymerase binding sites. SP6 RNA polymerase was used for the sense probe and T7 RNA polymerase for the antisense probe. Primers are listed in Supplementary Table S1. Floral bud samples were incubated at 4 °C or 22 ± 1 °C in a culture room in the light, with light intensity of 100–120 μmol m^−2^ s^−1^, fixed in 3.7% FAA (3.7% formaldehyde, 5% glacial acetic acid, and 50% ethanol) overnight at 4 °C, dehydrated in a gradient, embedded, sectioned, subjected to *in situ* hybridization, and viewed under a microscope. Since the developmental stage of floral organs is difficult to determine through external observation of floral buds and can only be clearly identified during the sectioning process, all samples were incubated for the same duration.

### Observation of the adaxial and abaxial epidermis of normal petals and petaloid stamens

Central cells from both normal petals and petaloid stamens were used for observation (Supplementary Fig. S1). Petaloid stamens needed to be longer than 1.5 cm. The abaxial and adaxial epidermal cells of the normal petals and petaloid stamens were peeled off using tweezers and placed on a microscope slide. The cell tissues were spread out using deionized water, and the cell number and morphology were observed with a Zeiss LSM 800 microscope (Carl Zeiss, Oberkochen, Germany).

### RNA extraction and quantitative real-time PCR

Floral buds at stage 4 of development were incubated at 4 °C or 22 °C. Samples were frozen at –80 °C and ground in liquid nitrogen. Total RNA was extracted from the samples using an RNA extraction kit (TaKaRa, Ohtsu, Japan). Reverse transcription was performed using HiScript^®^ II Q RT SuperMix +gDNA wiper (Vazyme, Nanjing, China) using 2000 ng cDNA in a 40 μl reaction volume. After reverse transcription, 160 μl H_2_O was added to dilute the sample to 200 μl. Gene expression was detected in 1 μl cDNA using an M5 HiPer Real-time PCR mix (Mei5bio, Beijing, China) and the ABI StepOne system (Thermo Fisher Scientific). *RhUBI2* and *RhACTIN5* were used as reference genes (Chen *et al*., 2023). All of the above experiments consisted of at least seven biological and three technical replicates. The related primers are shown in Supplementary Table S1.

### Virus-induced gene silencing in *R. hybrida* cv. ‘Samantha’

Virus-induced gene silencing (VIGS) was performed as previously described (Tian *et al*., 2014). Specific fragments of *RhMYB17*, *RhAP2*, and *RhAP2L* were inserted into the TRV2 vector. The resulting plasmids were transformed into *Agrobacterium tumefaciens* strain GV3101, and the bacterial cultures were used to infect rose cuttings as described previously (Wu *et al*., 2017; Zhang *et al*., 2019; Chen *et al*., 2021). When a portion of the floral buds emerged and their length exceeded 2 mm (stage 4), the developmental stage of the buds was examined under a microscope, and RNA was extracted for quantitative real-time PCR (qRT–PCR) analysis. qRT–PCR was performed to identify control and *RhMYB17*-, *RhAP2*-, and *RhAP2L*-silenced plants based on *RhMYB17*, *RhAP2*, and *RhAP2L* expression. Phenotypes of control plants and *RhMYB17-*, *RhAP2-*, or *RhAP2L-*silenced plants were recorded 55–60 d after inoculation, and floral organs were counted after stage 10 of flower development. When flowers opened (stage 10), they were dissected, and the floral organs (petals, stamens, and pistils) in four whorls were counted. Petal/stamen chimeras, recorded as petaloid stamens when stamens develop into petals, have the main characteristic of retaining stamen features, leading to asymmetric petal growth. The total number of stamens included normal stamens and petaloid stamens. Primers used for VIGS are listed in Supplementary Table S1.

### Transcriptional activity assay

Transcriptional activity assays of *RhMYB17* were performed using two methods: a transactivation assay in yeast and a dual-luciferase reporter assay in tobacco. For the transactivation assay in yeast, pGBKT7-RhMYB17 and pGBKT7-VP16 vectors were constructed and transformed into yeast strain Y2HGold. pGBKT7-VP16 served as a positive control, while the empty vector pGBKT7 served as a negative control. Transformed yeast cultures were diluted 10-fold and plated on synthetic drop-out (SD) media (SD/–Trp, SD/–Trp–His or SD/–Trp–His+X-gal).

For the transactivation assay in tobacco, the full-length coding sequence of *RhMYB17* was cloned into the pBD-EAQ vector driven by the *35S* promoter. pBD-VP16 was used as the positive control and pBD-empty was used as the negative control. The effector was introduced into *Agrobacterium tumefaciens* strain GV3101 and co-infected into tobacco with a dual reporter vector containing *GAL4-LUC* and *REN* (as an internal control) driven by the *35S* promoter. All primers used for cloning are listed in Supplementary Table S1. LUC and REN luciferase activities were measured using a dual-luciferase assay kit (Promega). Analysis was performed using a Luminoskan Ascent Microplate Luminometer (Thermo Fisher Scientific) according to the manufacturer’s instructions. Results were obtained by calculating the ratio of LUC to REN activity. Each assay contained at least three measurements.

### Subcellular localization

The coding sequence of *RhMYB17* was fused to *GFP* and inserted into pSuper1300 (Gong *et al*., 2002). pSuper1300-NF-YA4-mCherry was used as a nucleus marker (Wu *et al*., 2017). The vectors were transformed into *A. tumefaciens* strain GV3101. *Agrobacterium* cells were grown, collected, and resuspended in a solution of 10 mM MES, 10 mM MgCl_2_, and 200 μM acetosyringone to OD_600_=1.0. *Agrobacterium* cultures were injected separately into tobacco leaves. After 3 d, the leaf epidermal cells were examined by confocal laser-scanning fluorescence microscopy (Olympus FluoView FV1000). pSuper1300-GFP, serving as a control, can be commonly observed by the widespread expression of green fluorescent protein (GFP) within the tobacco leaf cells, including the nuclei, cytoplasm, and cell membrane. The Subcellular localization analysis was performed as previously described (Zhang *et al*., 2018; Liu *et al*., 2019).

### Phylogenetic tree construction and sequence analysis

When constructing a phylogenetic tree, we not only included the differentially expressed MYB transcription factor genes identified in the transcriptome, but also incorporated genes related to the development of the shoot apical meristem and floral organs in Arabidopsis, in order to more precisely identify key genes affecting the development of floral organs in rose. Their sequences were aligned using MAFFT (https://mafft.cbrc.jp/alignment/software/) with default parameters (Rozewicki *et al*., 2019). The alignment results were used as input in IQ-TREE (http://www.iqtree.org/) (Minh *et al*., 2020), and the maximum-likelihood method was used to construct a phylogenetic tree with 1000 bootstrap replicates. The motifs of the amino acid sequences were obtained using MEME Suite (https://meme-suite.org/meme/tools/meme) (Bailey *et al*., 2015). The results were visualized with TBtools (Chen *et al*., 2020). *cis*-Acting elements involved in low-temperature responsiveness in the gene promoters were analysed using the PlantCARE database (http://bioinformatics.psb.ugent.be/webtools/plantcare/html/) (Lescot *et al*., 2002). The specific binding motif of AtMYB17 was identified in the JASPAR-2022 database (https://jaspar.genereg.net/) (Castro-Mondragon *et al*., 2022); we reasoned that RhMYB17 has the same binding site. All gene promoters were scanned for potential binding sites using Find Individual Motif Occurrences (FIMO) (https://meme-suite.org/meme/tools/fimo) (Grant *et al*., 2011).

### Yeast one-hybrid assay

The *RhMYB17* coding sequence was cloned into the pGADT7 vector (Takara Bio, Mountain View, CA, USA) under the control of the *Galactokinase 4* (*GAL4*) promoter to create the effector constructs. The 2-kb sequences before the start codons were used as the promoters to construct the pHIS2 vectors. The promoters (*ProRhAG*, *ProRhMADS6-1*, *ProRhAP2*, and *ProRhAP2L*) were located upstream of the *LacZ* reporter gene (BD Biosciences, Shanghai, China). The resulting pHIS2 vectors were transformed into yeast strain Y187 for experimental verification. Self-activation of the promoters was suppressed using 3-amino-1,2,4-triazole. The yeast one-hybrid (Y1H) assay was performed as previously described (Wang *et al*., 2018).

### Dual-luciferase reporter assay

LUC/REN activity was determined as previously described (Gao *et al*., 2019). The promoter fragments of *RhAP2* and *RhAP2L* were inserted into the pGreenII0800-LUC vector as reporter constructs (Hellens *et al*., 2005). The coding sequence of *RhMYB17* was inserted after the *35S* promoter in the pGreenII62-SK vector as the effector. The recombinant plasmids were transformed into *Agrobacterium* (GV3101) and introduced into tobacco leaves by injection. Following 3 d of incubation, luminescence was detected with a live imaging apparatus and measured using the E1910 Dual-Luciferase Reporter Assay System (Promega) (Liang *et al*., 2020).

### Electrophoretic mobility shift assay

The complete coding sequence of *RhMYB17* was cloned into pET28a (6×His) (Scheurer *et al*., 2000) for prokaryotic expression of the fusion protein. The newly constructed vector was transformed into *Escherichia coli* strain Rosetta. To induce protein expression, 0.4 mM isopropyl β-d-1-thiogalactopyranoside was added to *E. coli* cultures in LB medium, followed by incubation at 16 °C for 12 h. The *E. coli* cells were centrifuged at 4000 *g* at 4 °C for 12 min, and the LB medium was removed. The *E. coli* cells were resuspended in phosphate buffered saline (PBS) for ultrasonic cell disruption. Before disruption, 1 mM phenylmethanesulfonyl fluoride (PMSF) and protease inhibitor cocktail (Roche, Basel, Switzerland) were added. The RhMYB17–His protein was purified with Ni Sepharose 6 Fast Flow (Cytiva, Marlborough, MA, USA). The RhMYB17–His protein was bound to the beads using the binding buffer (PBS supplemented with 10 mM imidazole, 5% glycerol, 1 mM PMSF, and protease inhibitor cocktail). The RhMYB17–His protein was then eluted using a concentration of 100 mM imidazole. The probes were labeled with biotin and annealed to form double-stranded DNA using the Annealing Buffer for DNA Oligos (Beyotime Biotechnology, Shanghai, China). For electrophoretic mobility shift assay (EMSA) experiments, binding reactions required the addition of 50 ng μl^−1^ poly(dI-dC), 5 mM MgCl_2_, 2.5% glycerol, and 0.05% NP-40 to the binding buffer. The concentration of the RhMYB17–His protein was maintained at 1 mg ml^−1^, and 2 µl was added to each experimental group. The biotin-labeled probe concentration was set at 20 fmol. EMSAs were performed with the LightShift chemiluminescent EMSA kit (Thermo Fisher Scientific) according to the manufacturer’s instructions.

### Transcriptome sequencing and differential gene expression analysis

The low-temperature transcriptome samples were from stage 4 floral buds treated at 4 °C for 1 d. Meanwhile, floral buds at stage 4 from VIGS were subjected to transcriptome sequencing. For each sample, 1.5 µg RNA was used as input material for rRNA removal using a RiboZero rRNA Removal Kit (Epicentre, Madison, WI, USA). Six sequencing libraries were generated using a NEBNext Ultra Directional RNA Library Prep Kit (NEB, Ipswich, MA, USA), following the manufacturer’s recommendations. The libraries were sequenced on the Illumina NovaSeq 6000 platform (Illumina, San Diego, CA, USA), and paired-end reads were generated. Differential expression analysis of two groups of floral buds (three biological replicates per group) was performed using the DESeq2 R package (Anders and Huber, 2010). Genes with an adjusted *P* value <0.05 and an absolute value of log_2_(fold-change)>1 were designated differentially expressed genes (DEGs).

### Statistical analysis

Statistical analysis was performed using SPSS Statistics software (IBM Corp., Armonk, NY, USA) and GraphPad Prism 8.4.3 (GraphPad Software Inc., San Diego, CA, USA). Data were analysed using one-way analysis of variance (ANOVA) followed by Tukey’s multiple range test to compare differences among samples at *P*<0.05. Statistically significant differences (*P<0.05, **P<0.01, and ***P<0.001) were determined by Student’s *t*-test.

### Accession numbers

Rose gene sequences can be found in the *Rosa chinensis* ‘Old Blush’ genome portal (https://lipm-browsers.toulouse.inra.fr/pub/RchiOBHm-V2) (Raymond *et al*., 2018). Arabidopsis gene sequences can be found in the Arabidopsis Genome TAIR database (https://www.arabidopsis.org). Accession numbers used in this study are as follows: *RhMYB17* (RchiOBHmChr1g0354061), *RhAP2* (RchiOBHmChr2g0106221), *RhAP2L* (RchiOBHmChr3g0468481), *RhAG* (RchiOBHmChr5g0012431), *RhUBI2* (RchiOBHmChr1g0359561), *RhACTIN5* (RchiOBHmChr3g0466761), *AtMYB17* (AT3G61250), *AtMYB13* (AT1G06180), *AtMYB16* (AT5G15310), *AtMYB56* (AT5G17800), and *AtMYB117* (AT1G26780).

## Results

### *RhMYB17* is induced by low temperature

**Fig. 1.**
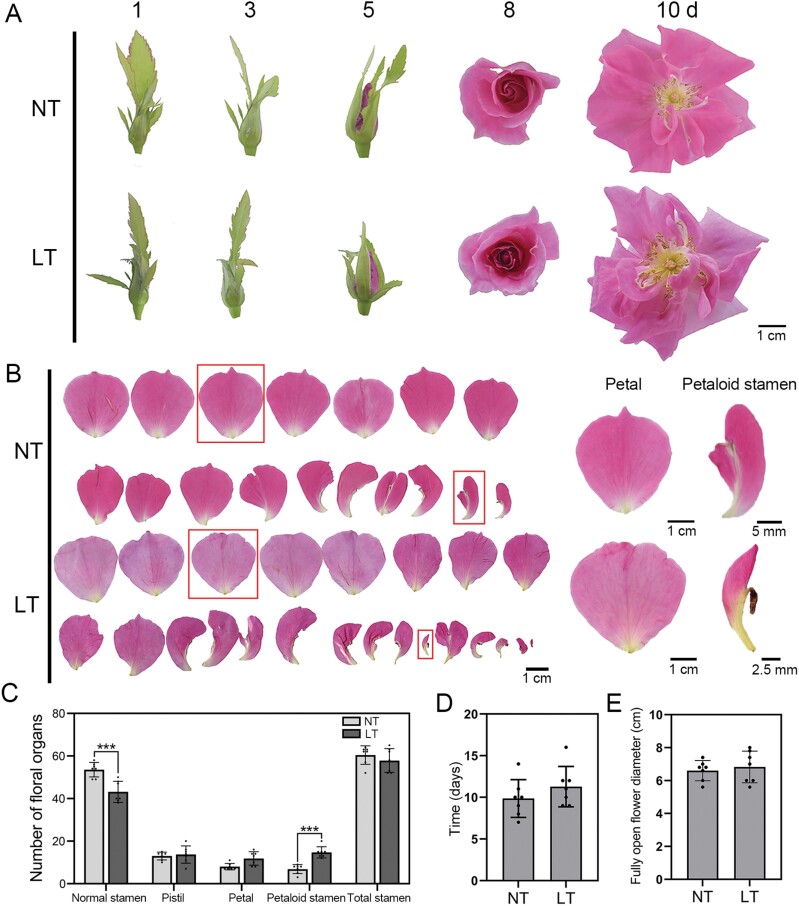
Low temperature affects the homeotic transformation of floral organs in rose. (A) Stage 4 floral buds exhibited flower opening phenotypes after 7 d treatment at low temperature (LT, 4 °C). These phenotypes were recorded at 1, 3, 5, 8, and 10 d, starting from stage 6 of flower development. Roses grown at the normal temperature (NT, 22 ± 1 °C) were used as a control to compare with the experimental group. Scale bar, 1 cm. (B) Normal petal and petaloid stamen phenotypes of NT and LT plants. The images on the right are close-ups of the petals in the red frames in the left panel. (C) Bar graph of the number of floral organs in NT and LT plants. Total petal number includes normal petals and petaloid stamens. Mean values ±SD are shown from seven biological replicates (*n*=7). Asterisks represent statistically significant differences (***P<0.001), as determined by Student’s *t*-test. (D) The time (d) taken for fully developed buds to bloom (from stage 6 to stage 10) in NT and LT plants. Mean values ±SD are shown from seven biological replicates (*n*=7). (E) Diameter of fully open flowers in NT and LT plants. Mean values ±SD are shown from seven biological replicates (*n*=7).

Low temperature significantly increases petaloid stamen number in rose (*R. hybrida* cv. ‘Vendela’) flowers (Ma *et al*., 2015). Based on previous studies, we divided floral bud development and blooming into 11 stages (Supplementary Table S2). To investigate the mechanism responsible for increased petaloid stamen number in rose under low-temperature conditions, after identifying the floral organs (stage 4), we kept floral buds (*R. hybrida* cv. ‘Samantha’) at 4 °C for 7 d. We detected a marked increase in petaloid stamen number accompanied by a significant reduction in normal stamen number following cold treatment (Fig. 1A–C). Close-up pictures further displayed the characteristics of petals and petaloid stamens, clearly revealing that the petaloid stamen exhibited characteristics of both petal and stamen (Fig. 1B, right). Concurrently, the petals exhibited larger abaxial and adaxial epidermal cell sizes compared with the petaloid stamens. However, there was no discernible difference in cell morphology (Supplementary Fig. S2). We observed an increased number of petaloid stamens, along with the transformation of some stamens into petals (Fig. 1C). Low-temperature treatment did not significantly affect the blooming time (from stage 6 to stage 10) of the floral buds (Fig. 1D). At stage 10, the flower diameter at full bloom in low-temperature-treated floral buds was comparable to that of the control (Fig. 1E). These results showed that under the cold stress, the development of petals and stamens was seriously affected.

**Fig. 2.**
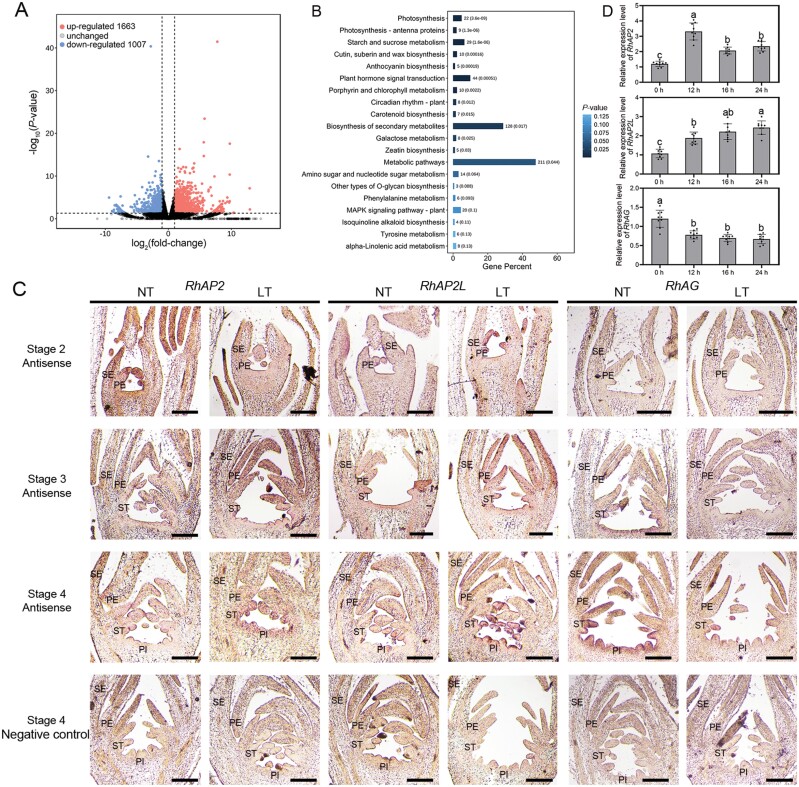
Transcriptome analysis of floral buds under normal temperature (NT) and low temperature (LT) conditions. (A) Volcano plot displaying the differentially expressed genes (DEGs) in floral buds that respond to LT. (B) KEGG pathway enrichment of DEGs in floral buds that respond to LT. (C) *In situ* hybridization of *RhAP2*, *RhAP2L*, and *RhAG* in floral buds at stages 2, 3, and 4 under NT and LT conditions. The sense probe was used as a negative control. Images show vertical sections of floral buds. Scale bar, 200 µm. PE, petal; PI, pistil; SE, sepal; ST, stamen. (D) Analysis of the expression levels of *RhAP2*, *RhAP2L*, and *RhAG* in floral buds at LT by qRT-PCR. Stage 4 floral buds were cultured at 4 °C for 0, 12, 16, and 24 h and subjected to gene expression analysis. Mean values ±SD are shown from nine biological replicates (*n*=9). *RhUBI2* and *RhACTIN5* were used as internal reference genes. Different letters above the bars indicate significantly different values (*P*<0.05) calculated using one-way ANOVA followed by a Tukey’s multiple range test.

A previous study indicated that short-term low-temperature stress affects the expression of MIKC^C^-type MADS box genes, with *RhAP1* and *RhFUL* (class A genes) significantly up-regulated under cold stress (Wang *et al*., 2022). Simultaneously, low temperature stress leads to the activation and inhibition of transcription factors within a few hours (Jia *et al.*, 2016; Zhao *et al.*, 2016). Therefore, we chose flower buds subjected to 24 h of low temperature treatment for transcriptome sequencing to identify genes involved in the low-temperature response in flower buds. To further explore the effects of cold stress on the homeotic transformation of floral organs, we identified 2670 DEGs in cold-treated samples versus the control, including 1663 up-regulated and 1007 down-regulated genes (Fig. 2A; Supplementary Tables S3, S4). Kyoto Encyclopedia of Genes and Genomes (KEGG) pathway enrichment analysis revealed that terms such as ‘Plant hormone signal transduction’, ‘Biosynthesis of secondary metabolites’, and ‘Metabolic pathways’ were enriched among the DEGs (Fig. 2B). *In situ* hybridization results for floral buds at various developmental stages indicated that *RhAP2*, *RhAP2L*, and *RhAG* were distinctly expressed at stage 4. *RhAP2* and *RhAP2L* were expressed in the primordia of petals, stamens, and pistils, while *RhAG* was expressed in the primordia of stamens and pistils (Fig. 2C). The expression levels of certain floral organ identity genes were significantly altered in floral buds (stage 4) kept at low temperature (4 °C) for 24 h (Fig. 2D).

**Fig. 3.**
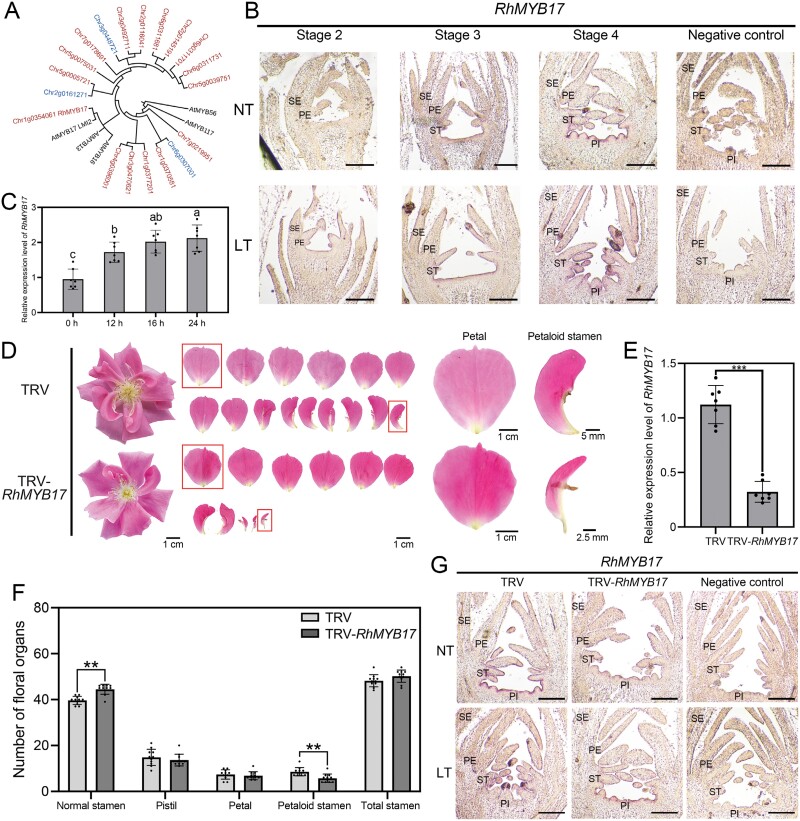
*RhMYB17* expression increases under low temperature (LT) conditions and is involved in governing the homeotic transformation of floral organs. (A) Phylogenetic tree of potential MYB transcription factors in response to LT. Red font represents up-regulated genes and blue font represents down-regulated genes. (B) *In situ* hybridization of *RhMYB17* in floral buds at stages 2, 3, and 4 under normal temperature (NT) and LT conditions. Scale bar, 200 µm. PE, petal; PI, pistil; SE, sepal; ST, stamen. (C) *RhMYB17* expression in stage 4 floral buds at 0, 12, 16, and 24 h under LT conditions. 0 h represents the stage 4 flower buds without treatment with low temperature, as a control. Mean values ±SD are shown from seven biological replicates (*n*=7). *RhUBI2* and *RhACTIN5* were used as internal reference genes. Different letters above the bars indicate significantly different values (*P*<0.05) calculated using one-way ANOVA followed by a Tukey’s multiple range test. (D) Normal petal and petaloid stamen phenotypes of TRV and TRV-*RhMYB17* plants. The images on the right are close-ups of the petals in the red frames in the left panel. The plants were cultured at a normal temperature. (E) Relative expression of *RhMYB17* in TRV and TRV-*RhMYB17* plants. Mean values ±SD are shown from seven biological replicates (*n*=7). Asterisks represent statistically significant differences (***P<0.001), as determined by Student’s *t*-test. (F) Bar graph of the number of floral organs in TRV and TRV-*RhMYB17* plants. Total petal number includes normal petals and petaloid stamens. The plants were cultured at a normal temperature. Mean values ±SD are shown from eleven biological replicates (*n*=11). Asterisks represent statistically significant differences (**P<0.01), as determined by Student’s *t*-test. (G) *In situ* hybridization of TRV and TRV-*RhMYB17* during early floral bud development (stage 4) under NT and LT conditions. The sense probe (SP6) was used as a negative control. Images show vertical sections of floral buds. Scale bar, 200 µm. Abbreviations as for (B).

Transcription factor genes constituted 6.55% (175) of the DEGs identified, including 30 down-regulated and 145 up-regulated genes (Supplementary Fig. S3). Among the differentially expressed transcription factor genes, MYB transcription factors represented the highest proportion (Supplementary Table S5). We used the protein sequences of these MYB transcription factors, along with those involved in the development of the shoot apical meristem and floral organs in Arabidopsis, to construct a phylogenetic tree (Fig. 3A). It is clearly observable that Chr1G0354061 clusters together with AtMYB17, AtMYB13, and AtMYB16, whereas other MYB transcription factors, which are differentially expressed under cold treatment, do not cluster with those of Arabidopsis. Meanwhile, AtMYB17 is able to regulate the expression of a class A gene (*AtAP1*), thereby controlling the development of floral organs in Arabidopsis. Therefore, we have selected Chr1G0354061 for subsequent research.

We identified a MYB transcription factor gene (*Chr1G0354061*) that was up-regulated under low-temperature conditions and that was homologous to *AtMYB17*, which we named *RhMYB17*. We assessed the expression level of *RhMYB17* in floral buds following low-temperature treatment using qRT-PCR. *In situ* hybridization results confirmed that under both low-temperature and normal conditions, *RhMYB17* is expressed in the stamen and pistil primordia of floral buds. A distinct expression signal of *RhMYB17* was observed in the stage 4 floral buds (Fig. 3B). We measured the expression levels of *RhMYB17* in flower buds at different times of low-temperature treatment. During the low-temperature treatment of stage 4 floral buds, the expression of *RhMYB17* was significantly increased at 12, 16, and 24 h (Fig. 3C). These results suggest that low temperatures can enhance the expression of *RhMYB17* in floral buds.

### RhMYB17 regulates the homeotic transformation of floral organs

To elucidate the role of RhMYB17 in floral organ development, we employed VIGS to silence *RhMYB17* in rose plants. Silencing *RhMYB17* reduced the number of petaloid stamens and increased the number of normal stamens (Fig. 3D), indicating that *RhMYB17* is crucial for the homeotic transformation of floral organs and that a reduction in its expression results in aberrant floral organ development. Close-up images revealed that the petaloid stamens exhibit stamen characteristics (Fig. 3D, right). The expression of *RhMYB17* in silenced plants was significantly lower compared with the control, indicating a high level of silencing efficiency (Fig. 3E). We observed a reduction in the number of petaloid stamens in *RhMYB17*-silenced plants compared with the control (Fig. 3F). The development of petaloid stamens was relatively delayed, and their abaxial and adaxial epidermal cells were notably smaller than those of petals (Supplementary Fig. S4). To observe the changes in gene expression in response to low temperature in *RhMYB17*-silenced plants more directly, we performed qRT-PCR analysis on stage 4 floral buds. Although low temperature promoted *RhMYB17* expression in *RhMYB17*-silenced plants, its expression level remained lower than it was in the control (Supplementary Fig. S5). Meanwhile, *in situ* hybridization results further revealed that the expression of *RhMYB17* was located on the primordia of stamens and pistils, and this distribution of expression was not altered by cold treatment (Fig. 3G).

### RhMYB17 localizes to the nucleus and functions as a transcriptional activator

**Fig. 4.**
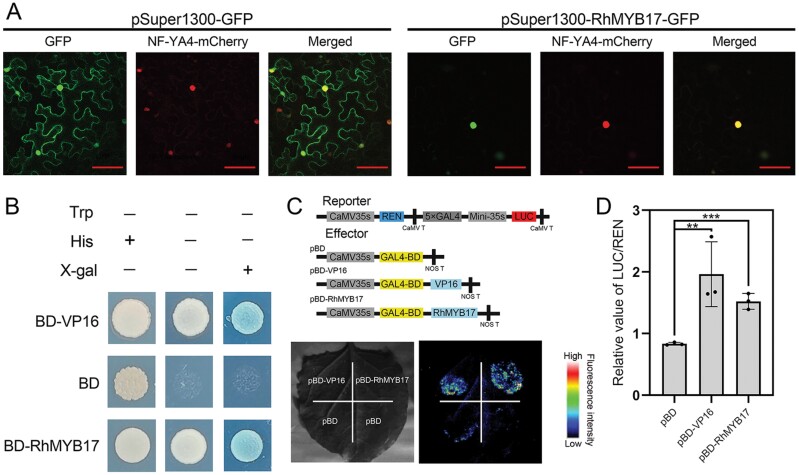
Characteristics of RhMYB17. (A) Subcellular localization of heterologously expressed RhMYB17 in tobacco leaves. NF-YA4-mCherry red fluorescence was used as a nucleus marker. Scale bar, 50 µm. (B) Transcriptional activator activity of RhMYB17 in yeast. BD and BD-VP16 were used as negative and positive controls, respectively. The transformants were streaked onto SD/–Trp, SD/–Trp–His, and SD/–Trp–His+X-gal plates and incubated at 30 °C for 3 d. β-Galactosidase activity was examined by X-gal staining. (C) Transcriptional activator activity of RhMYB17 in tobacco leaf epidermal cells. Upper panel, schematic representation of the reporter and effector constructs. Lower panel, live imaging of the transcriptional activator activity of RhMYB17. pBD and pBD-VP16 were used as negative and positive controls, respectively. (D) Quantitative analysis of the transcriptional activator activity of RhMYB17. The mean values ±SD are shown from three biological replicates (*n*=3). Asterisks represent statistically significant differences (**P<0.01, ***P<0.001), as determined by Student’s *t*-test.

RhMYB17 is a putative R2R3-MYB transcription factor. Subcellular localization analysis revealed that RhMYB17 localized to the nucleus, where it functioned as a transcription factor involved in gene regulation. Fluorescence from the vector pSuper1300-GFP reporter used as a control can commonly be observed anywhere in the cell, as GFP itself does not have subcellular localization specificity. (Fig. 4A).

We conducted transcriptional activity assays of *RhMYB17* using two methods: a transactivation assay in yeast and a dual-luciferase reporter assay in tobacco. When we cloned *RhMYB17* in pGBKT7, the resulting construct (pGBKT7-RhMYB17) was fully activated in yeast cells (Fig. 4B). In a dual-luciferase reporter assay, pBD-RhMYB17 exhibited significantly higher LUC activity compared with pBD alone when transiently expressed in tobacco leaf epidermal cells (Fig. 4C), indicating that RhMYB17 acts as a transcriptional activator. Analysis of the LUC/REN ratio indicated that pBD-RhMYB17 had higher LUC activity than did the pBD control (Fig. 4D).

### Transcriptome analysis of potential regulatory pathways involving RhMYB17

**Fig. 5.**
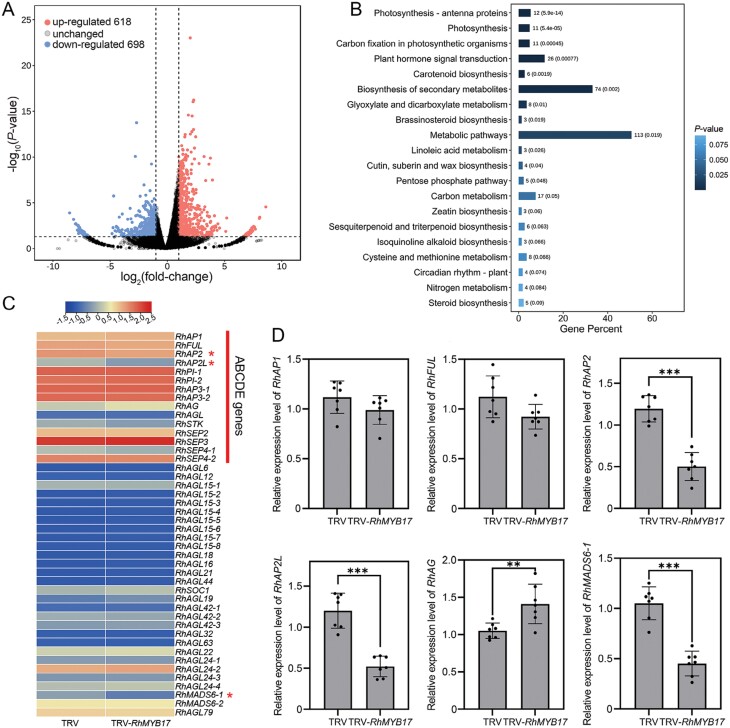
Transcriptome analysis of the potential regulatory pathways of RhMYB17. (A) Volcano plot displaying the differentially expressed genes (DEGs) in TRV-*RhMYB17* versus TRV plants. (B) KEGG pathway enrichment of DEGs in TRV and TRV-*RhMYB17* plants. (C) Fragments per kilobase per million mapped reads (FPKM) values of *RhAP2*, *RhAP2L*, and the MIKC^C^-type MADS box genes in TRV and TRV-*RhMYB17* plants visualized in a heatmap. Red asterisks indicate significantly differentially expressed genes. (D) Relative expression of *RhAP1*, *RhFUL*, *RhAP2*, *RhAP2L*, *RhAG*, and *RhMADS6-1* in TRV and TRV-*RhMYB17* plants, as determined by qRT-PCR. Mean values ±SD are shown from seven biological replicates (*n*=7). Asterisks represent statistically significant differences (**P<0.01, ***P<0.001), as determined by Student’s *t-*test.

To further elucidate the molecular function of RhMYB17, we conducted transcriptome sequencing of floral buds in which *RhMYB17* was transiently silenced by VIGS. We identified 1316 DEGs, among which 618 were up-regulated and 698 were down-regulated in *RhMYB17*-silenced plants versus the control (Fig. 5A; Supplementary Tables S6, S7). KEGG pathway enrichment analysis revealed that terms such as ‘Plant hormone signal transduction’, ‘Biosynthesis of secondary metabolites’, and ‘Metabolic pathways’ were enriched among the DEGs (Fig. 5B). We speculate that RhMYB17 may mediate plant hormone signal transduction and consequently influence the homeotic transformation of floral organs under low-temperature conditions. Furthermore, *RhAP2*, *RhAP2L*, and *RhMADS6-1* expression was significantly reduced in *RhMYB17*-silenced plants versus the control, as shown in the fragments per kilobase per million mapped reads (FPKM) heatmap (Fig. 5C). Finally, we confirmed the expression patterns of key genes by qRT-PCR. *RhAP2*, *RhAP2L*, and *RhMADS6-1* were significantly down-regulated, whereas *RhAG* was up-regulated, in *RhMYB17*-silenced plants versus the control. The relative expression levels of *RhAP1* and *RhFUL* (class A genes), which are responsive to cold stress, were also detected in *RhMYB17*-silenced plants versus the control. No significant differences were observed, indicating that RhMYB17 does not participate in the regulation of their expression (Fig. 5D). Combined analysis of the *RhMYB17*-silenced transcriptome and the low-temperature transcriptome revealed the differential expression of genes related to floral organ development. Among these, *RhAP2*, *RhAP2L*, *RhAG*, and *RhMADS6-1* may participate in the homeotic transformation of floral organs and are regulated by RhMYB17.

### RhMYB17 directly binds to the promoters of *RhAP2* and *RhAP2L* to regulate their expression

**Fig. 6.**
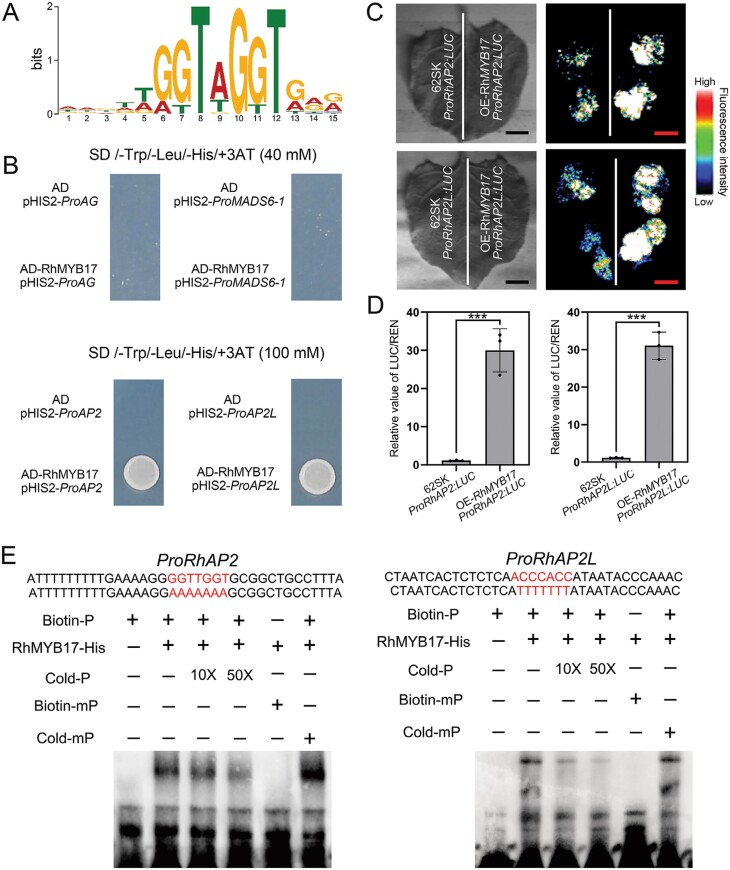
*RhAP2* and *RhAP2L* are direct target genes of RhMYB17. (A) The specific binding motif of RhMYB17 predicted from homologous proteins. Letter size represents the probability that the base is present in the binding site. (B) Yeast one-hybrid analysis to detect interactions between RhMYB17 and the promoters of *RhAG*, *RhMADS6-1*, *RhAP2*, and *RhAP2L*. The three columns in the figure represent three independent repetitions. (C) Effect of RhMYB17 on activation of the *RhAP2* and *RhAP2L* promoters in tobacco leaves, as determined by measuring LUC/REN activity. The experimental group and the control group (empty vector, 62SK) were tested on the same tobacco leaves to ensure the consistency of the experimental data. Scale bar, 1 cm. (D) The ratio of LUC/REN in leaves expressing the empty vector (62SK) plus promoter was used for normalization (set to 1). Mean values ±SD are shown from three biological replicates (*n*=3). Asterisks represent statistically significant differences (***P<0.001), as determined by Student’s *t-*test. (E) Verification by EMSA that RhMYB17-His directly binds to the promoters of *RhAP2* and *RhAP2L*. Both the biotin probe (Biotin-P) and mutated probe (Biotin-mP) are shown.

RhMYB17 possesses a protein sequence homologous to AtMYB17, suggesting that RhMYB17 and AtMYB17 might share the same binding motifs. We identified the binding motifs of AtMYB17 using the JASPAR database (https://jaspar.genereg.net/). AtMYB17 specifically binds to the GGTA/T/GGGT binding sites in the promoters of its target genes (Fig. 6A). Consequently, we used the FIMO tool (https://meme-suite.org/meme/tools/fimo) to analyse the binding of all gene promoters in the rose genome. We determined that both *ProRhAP2* and *ProRhAP2L* possess specific binding sites.

We performed a Y1H analysis to validate the binding sites; *ProRhMADS6-1* was validated as a potential target gene based on the *RhMYB17*-silenced transcriptome; *ProRhAG* served as a control. In the Y1H assay, *ProRhAP2* and *ProRhAP2L* were activated by RhMYB17, whereas *ProRhAG* and *ProRhMADS6-1* were not (Fig. 6B). Dual-luciferase reporter assays revealed that *ProRhAP2* and *ProRhAP2L* were activated in *RhMYB17-*overexpressing tobacco plants versus the control (Fig. 6C, D). Finally, we generated biotin-labeled probes corresponding to fragments containing the GGTA/T/GGGT site in *ProRhAP2* (−1398 to −1404 bp) and *ProAP2L* (−1074 to −1068 bp) and used them in EMSAs. Unlabeled cold probe, but not unlabeled mutated cold probe, competed for RhMYB17–His binding with the biotin-labeled probe (Fig. 6E). These results indicate that RhMYB17 specifically binds to GGTA/T/GGGT motifs in the *RhAP2* and *RhAP2L* promoters.

### *RhAP2* or *RhAP2L* silencing reduces the number of petaloid stamens in rose

**Fig. 7.**
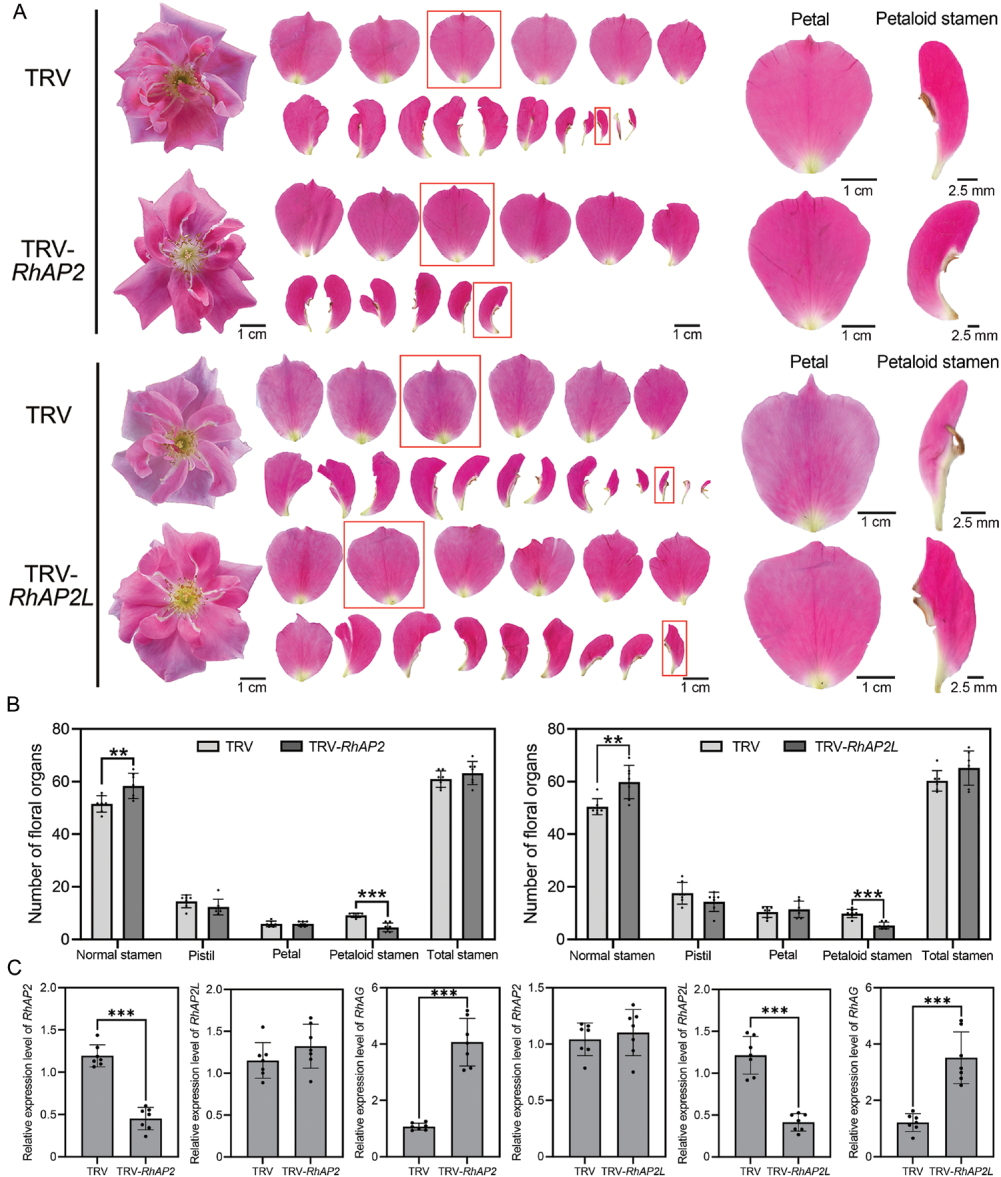
Verification of the roles of RhAP2 and RhAP2L in governing the homeotic transformation of floral organs by virus-induced gene silencing. (A) Normal petal and petaloid stamens phenotypes in TRV and TRV-*RhAP2* plants, as well as in TRV and TRV-*RhAP2L* plants. The images on the right are close-ups of the petals in the red frames in the left panel. The plants were cultured at a normal temperature. (B) Bar graph of the number of floral organs in both TRV and TRV-*RhAP2* plants, as well as in TRV and TRV-*RhAP2L* plants. Total petal number includes normal petals and petaloid stamens. Mean values ±SD are shown from seven biological replicates (*n*=7). (C) Relative expression levels of *RhAP2*, *RhAP2L*, and *RhAG* in TRV and TRV-*RhAP2* plants, as well as in TRV and TRV-*RhAP2L* plants, as determined by qRT-PCR. Mean values ±SD are shown from seven biological replicates (*n*=7). To ensure the stability of the experimental results, the control groups of the two genes were analysed independently. The control group and the experimental group were placed in the same culture environment. *RhUBI2* and *RhACTIN5* were used as internal reference genes. Asterisks in (B, C) represent statistically significant differences (**P<0.01, ***P<0.001), as determined by Student’s *t*-test.

To investigate the roles of RhAP2 and RhAP2L in the homeotic transformation of floral organs, we constructed TRV-*RhAP2* and TRV-*RhAP2L* vectors for VIGS in rose plants. Due to the high sequence similarity between *RhAP2* and *RhAP2L*, we specifically silenced the 5ʹ untranslated region of these genes. We observed a greater reduction in the number of petaloid stamens in TRV-*RhAP2* and TRV-*RhAP2L* plants, while the normal stamen number increased. These results suggest that *RhAP2* and *RhAP2L* influence the development of floral organs (Fig. 7A, B). There was no significant difference in abaxial and adaxial epidermal cell morphology. However, petaloid stamens exhibited smaller abaxial and adaxial epidermal cell sizes (Supplementary Fig. S6).

The expression levels of *RhAP2* and *RhAP2L* were significantly reduced in *RhAP2*- and *RhAP2L*-silenced plants compared with the TRV controls (Fig. 7C), indicating high silencing efficiency. *RhAG* (a class C gene) was differentially expressed in *RhAP2*- and *RhAP2L*-silenced plants compared with the TRV control (Fig. 7C). We speculated that when *RhAP2* and *RhAP2L* were silenced, increased ectopic expression of *RhAG* in the outer whorls led to a reduction in the number of petaloid stamens.

## Discussion

### The influence of low temperature on the homeotic transformation of floral organs in rose

Floral organ development is a critical stage for plants, particularly ornamental horticultural crops. It is influenced by various external factors, including temperature, light, and moisture. Low temperatures trigger flowering in ‘Hass’ avocado (*Persea americana* Mill.) (Acosta-Rangel *et al*., 2021). Low-temperature treatment alters certain floral characteristics in tuberose (*Agave amica*): storage at 12 °C delays the meristem transition, germination, and flowering time (Fragoso-Jimenez *et al*., 2021). Furthermore, low temperatures can affect flower development, delaying floral bud germination in various plants, such as rose, lily (*Lilium hansonii*), and chrysanthemum (*Dendranthema morifolium*) (Cockshull *et al*., 1981; Shin *et al*., 2000; Lucidos *et al*., 2013). Low temperature can induce homeotic transformation of floral organs, leading to morphological transformations and quantitative changes in floral organs among different floral whorls.

In this study, we exposed floral buds of *R. hybrida* cv. ‘Samantha’ to 4 °C for 7 d (after floral organ identity; stage 4) and transferred them to normal growth conditions. Compared with the control group, there was a significant increase in the number of petaloid stamens (Fig. 1), which is consistent with previous research findings (Ma *et al*., 2015; Han *et al*., 2018). We observed that the transformation of stamens into petals, which occurs after the floral organ identity-determining period (stage 4), is significantly enhanced under low-temperature treatment, leading to an increase in petaloid stamens. These findings suggest that multiple pathways regulate petal number. Additional research is needed to investigate the specific and general pathways regulating this trait.

### RhMYB17 responds to low temperature and activates the expression of *RhAP2* and *RhAP2L*

R2R3-MYB transcription factors, the most abundant group in the MYB transcription factor family (Dubos *et al*., 2010), regulate specific physiological and biochemical processes in plants. Various R2R3-MYB transcription factors actively participate in plant growth and development, secondary metabolism, and biotic and abiotic stress responses (Dubos *et al*., 2010; Ambawat *et al*., 2013; Baldoni *et al*., 2015; Li *et al*., 2015; Li *et al*., 2019; Liu *et al*., 2021). At present, there is limited research on the role of R2R3-MYB transcription factors in floral organ development. MYB123 is involved in the regulation of seed development and heat stress in Arabidopsis and affects the transformation of stamens to petals in rose (Nesi *et al*., 2001; Jacob *et al*., 2021; Li *et al*., 2022). In Arabidopsis, *AtMYB17* is highly expressed in inflorescences, especially during early flower development. *AtMYB17* transcript levels increase during seed germination and are gradually concentrated in the shoot apex (Zhang *et al*., 2009), but the specific function of this gene is unclear.

Here, we demonstrated that RhMYB17 is involved in the homeotic transformation of floral organs under low-temperature conditions, as it is induced by low temperature, and plants with VIGS-induced *RhMYB17* silencing exhibited fewer petaloid stamens (Fig. 3). RhMYB17 directly binds to GGTA/T/GGGT motifs in the *RhAP2*/*RhAP2L* promoters and participates in the homeotic transformation of floral organs. RhMYB17 functions as a transcriptional activator to up-regulate the expression of *RhAP2* and *RhAP2L* (Fig. 6). The accumulation of class A transcripts in floral buds significantly restricts expression of the class C gene, resulting in the transformation of outer stamens into petals.

### Antagonistic roles of floral organ identity genes

The class C gene *AG* directly influences floral organ identity (Bowman *et al*., 1991; Robles and Pelaz, 2004; Liu *et al*., 2013; Tanaka *et al*., 2013; Yan *et al*., 2016). *RhAP2* and *RhAP2L*, as class A genes, can antagonize the expression of *RhAG*, thereby playing a role in the homeotic transformation of floral organs.

*RhAP2* and *RhAP2L* are class A genes that are expressed in floral buds (Fig. 2D). Interestingly, the expression pattern of the *AP2* genes undergoes changes during floral organ development. In stages 2 and 3, its expression can be detected on petals, but in stage 4, the *AP2* genes are expressed in stamens and carpels. This expression pattern may be due to the complete development of the outer whorls of floral organs (sepals and outer petals), which leads to the restriction of *AP2* gene expression to the inner whorls. Although the general conservation of AP2 is widely accepted, there are examples in which divergence has occurred. In petunia, PhAp2A does not have perianth identity control function, whereas Arabidopsis AP2 has such a function (Maes *et al.*, 2001). In apple, AP2 is capable of regulating flavonoid biosynthesis, confirming that the functions of AP2 vary among different species, along with its distinct expression patterns (Ding *et al.*, 2022). *RhAP2-*/*RhAP2L*-silenced plants showed a reduced number of petaloid stamens and an increased number of normal stamens compared with the wild type (Fig. 7C). In *ap2* mutants, the expression of *AtAG* expands to the outer two floral whorls, indirectly increasing the expression of *AtAG* (Huang *et al*., 2017). This is consistent with the increased *RhAG* expression in *RhAP2*-/*RhAP2L*-silenced plants.

Moreover, we also observed that the expression of anthocyanin biosynthesis genes *RhCHSa*, *RhF3’H*, *RhANS*, *RhUFGT*, and *RhMYB1* was increased in the stage 4 rose buds subjected to low-temperature treatment (Supplementary Fig. S7). Therefore, cold stress may promote the accumulation of anthocyanins in the early stage of floral buds in rose. However, when the low-temperature-treated flower buds fully open, the color of the petals is lighter than that of the control (Fig. 1). We speculate that there is a complex correlation between flower color and metabolism of anthocyanins, low temperature, as well as the developmental stages of flower.

## Conclusion

Our study uncovers a novel mechanism responsible for the increased number of petaloid stamens in rose flowers at low temperature. *RhMYB17* expression increases under low-temperature conditions. RhMYB17 activates *RhAP2* and *RhAP2L* expression, thereby effecting the homeotic transformation of floral organs. This study offers insights into the regulation of homeotic floral organ transformation in response to abiotic stress.

## Supplementary data

The following supplementary data are available at *JXB* online.

Fig. S1. Sampling sites of the abaxial and adaxial epidermis of normal petals and petaloid stamens for observing cell morphology and size under confocal microscopy.

Fig. S2. Images of the abaxial and adaxial epidermal cells of normal petals and petaloid stamens captured using confocal microscopy.

Fig. S3. Analysis of transcription factor genes in the LT transcriptome.

Fig. S4. Images of the abaxial and adaxial epidermal cells of normal petals and petaloid stamens from TRV and TRV-*RhMYB17* captured using confocal microscopy.

Fig. S5. The relative expression levels of *RhMYB17* in stage 4 rose floral buds of TRV and TRV-*RhMYB17* under normal- and low-temperature (1 d) conditions.

Fig. S6. Images of the abaxial and adaxial epidermal cells of normal petals and petaloid stamens from TRV, TRV-*RhAP2*, and TRV-*RhAP2L* captured using confocal microscopy.

Fig. S7. Expression levels of anthocyanin biosynthesis genes in the low-temperature transcriptome of floral buds.

Table S1. Primer sequences used in this study.

Table S2. The 11 stages of flower development in rose.

Table S3. Up-regulated DEGs in the low-temperature transcriptome.

Table S4. Down-regulated DEGs in the low-temperature transcriptome.

Table S5. All differentially expressed MYB transcription factor genes in the low-temperature transcriptome.

Table S6. Up-regulated DEGs in the TRV and TRV-*RhMYB17* transcriptomes.

Table S7. Down-regulated DEGs in the TRV and TRV-*RhMYB17* transcriptomes.

erae099_suppl_Supplementary_Tables_S1-S7

erae099_suppl_Supplementary_Figures_S1-S7

## Data Availability

The datasets generated and analysed during the current study are available in NCBI Sequence Read Archive database under BioProject (accession numbers: PRJNA804258 and PRJNA818810).
