# Supplementary material for: The transcription factor RhMYB17 regulates the homeotic transformation of floral organs in rose (Rosa hybrida) under cold stress
Source: J Exp Bot. 2024 Mar 7;75(10):2965–81. doi: 10.1093/jxb/erae099 (PMC11103112; doi:10.1093/jxb/erae099)
Supplement: erae099_suppl_Supplementary_Figures_S1-S7 [file erae099_suppl_supplementary_figures_s1-s7.pdf]

Supplementary Fig. S1

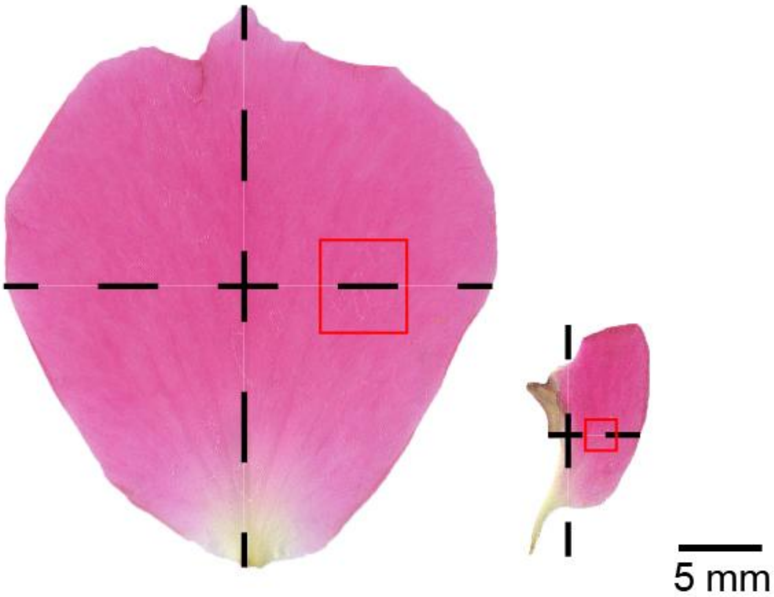

Supplementary Fig. S1. Sampling sites of the abaxial and adaxial epidermis of normal petals and petaloid stamens for observing cell morphology and size under confocal microscopy.

Supplementary Figure S2

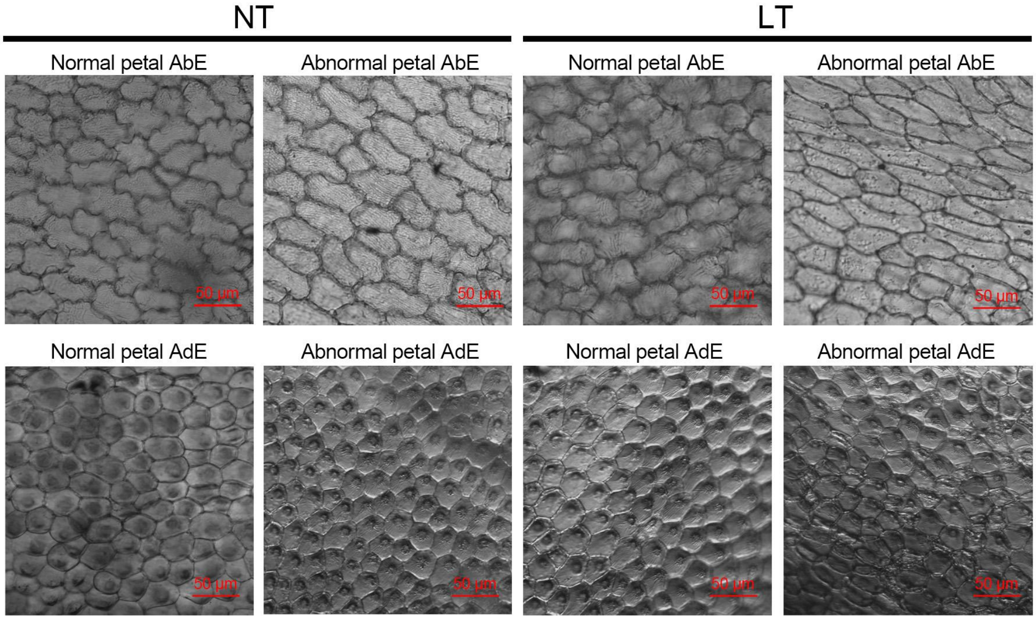

Supplementary Fig. S2. Images of the abaxial and adaxial epidermal cells of normal petals and petaloid stamens captured using confocal microscopy. LT represents low-temperature treatment (stage 4 rose flower buds exposed to 4°C for 7 d). The control is roses continuously cultivated under normal conditions (NT).

Supplementary Figure S3

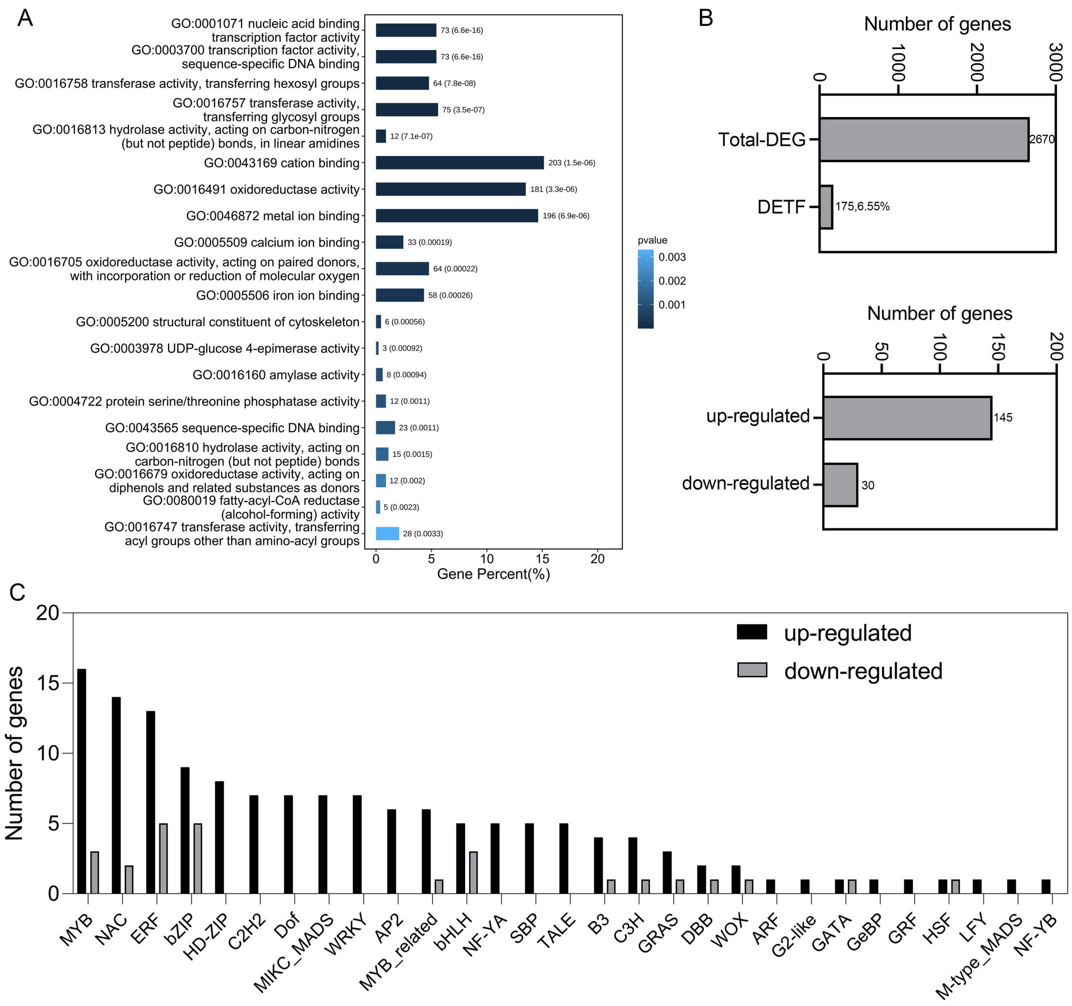

Supplementary Fig. S3. Analysis of transcription factor genes in the LT transcriptome. (A) Gene Ontology (GO) classification of the DEGs. (B) Differentially expressed transcription factor genes in the low-temperature transcriptome. (C) Proportion of DEGs in various transcription factor classes.

Supplementary Figure S4

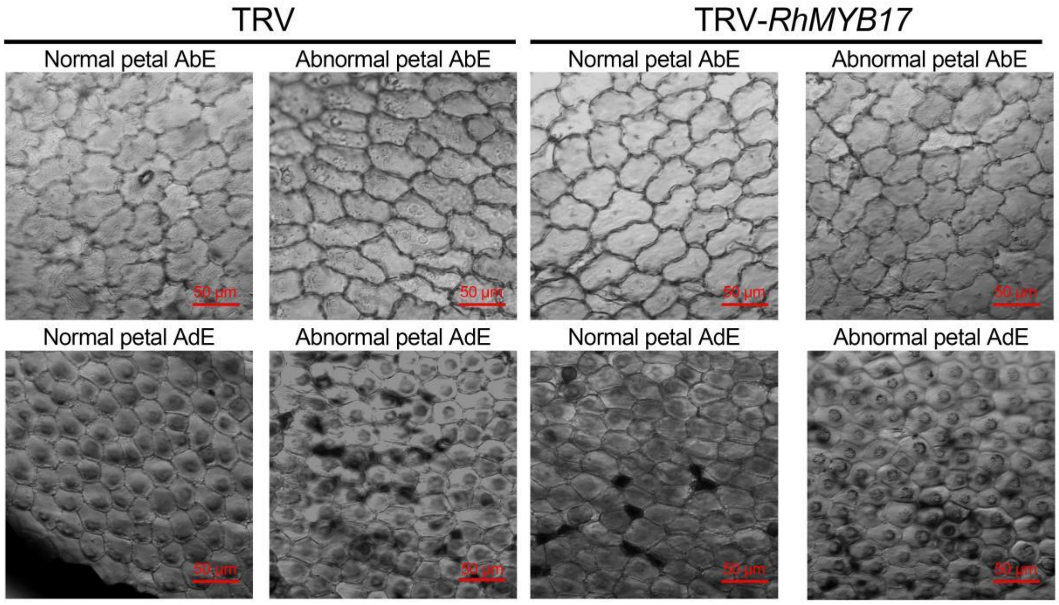

Supplementary Fig. S4. Images of the abaxial and adaxial epidermal cells of normal petals and petaloid stamens from TRV and TRV-*RhMYB17* captured using confocal microscopy.

Supplementary Figure S5

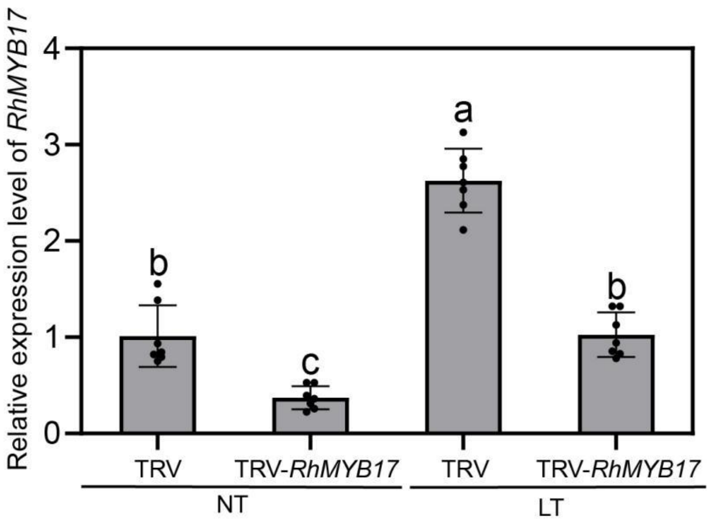

Supplementary Fig. S5. The relative expression levels of *RhMYB17* in stage 4 rose floral buds of TRV and TRV-*RhMYB17* under normal- and low-temperature (1 d) conditions.

Supplementary Figure S6

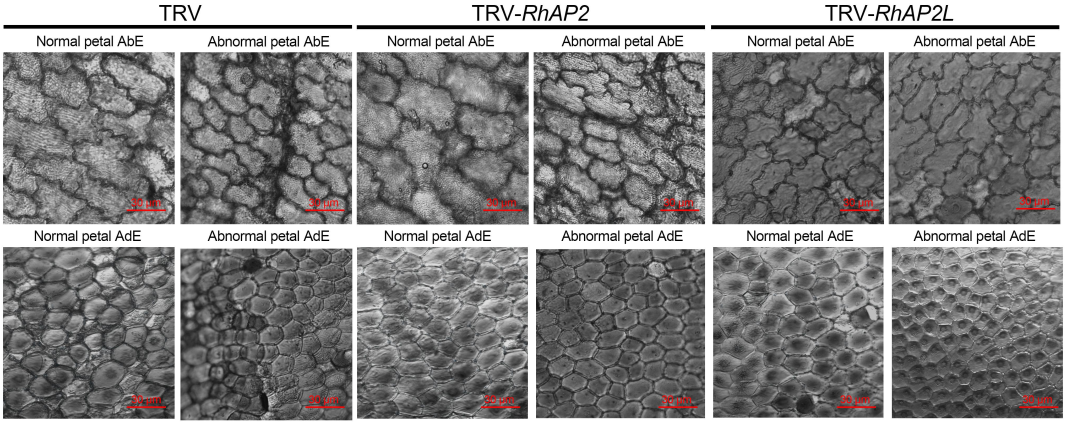

Supplementary Fig. S6. Images of the abaxial and adaxial epidermal cells of normal petals and petaloid stamens from TRV, TRV-*RhAP2*, and TRV-*RhAP2L* captured using confocal microscopy.

Supplementary Figure S7

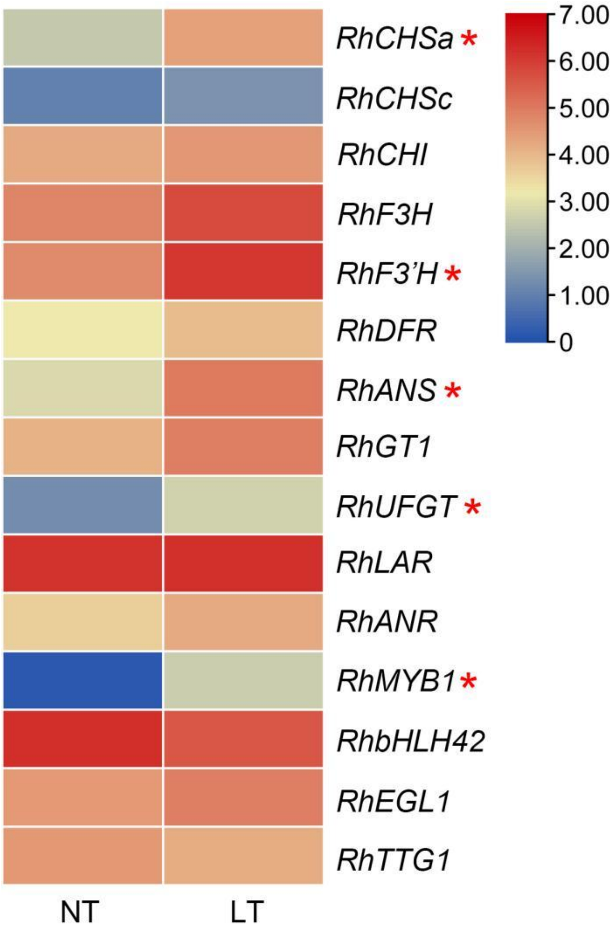

Supplementary Fig. S7. Expression levels of anthocyanin biosynthesis genes in the low-temperature transcriptome of floral buds. Genes with expression differences of more than 2-fold are indicated by red asterisks.
